# Identification of the difference in the pathogenesis in heart failure arising from different etiologies using a microarray dataset

**DOI:** 10.6061/clinics/2017(10)03

**Published:** 2017-10

**Authors:** Guodong Yang, Shuping Chen, Aiqun Ma, Jun Lu, Tingzhong Wang

**Affiliations:** I Department of Cardiovascular Medicine, First Affiliated Hospital of Xi’an Jiaotong University, China; II Key Laboratory of Molecular Cardiology, Shaanxi Province, China; III Key Laboratory of Environment and Genes Related to Diseases (Xi'an Jiaotong University), Ministry of Education, China; IV Clinical Research Center, First Affiliated Hospital of Xi’an Jiaotong University, China

**Keywords:** Heart Failure, Different Etiologies, Microarray, Expression Profile, Pathogenesis, Data Mining

## Abstract

**OBJECTIVES:**

Clinically, patients with chronic heart failure arising from different etiologies receive the same treatment. However, the prognoses of these patients differ. The purpose of this study was to elucidate whether the pathogenesis of heart failure arising from different etiologies differs.

**METHODS:**

Heart failure-related dataset GSE1145 was obtained from the Gene Expression Omnibus database. Differentially expressed genes were identified using R. A protein-protein interaction network of the differentially expressed genes was constructed using Search Tool for the Retrieval of Interacting Genes. The modules in each network were analyzed by Molecular Complex Detection of Cytoscape. The Database for Annotation, Visualization and Integrated Discovery was used to obtain the functions of the modules.

**RESULTS:**

Samples contained in GSE1145 were myocardial tissues from patients with dilated cardiomyopathy, familial cardiomyopathy, hypertrophic cardiomyopathy, ischemic cardiomyopathy, and post-partum cardiomyopathy. The differentially expressed genes, modules, and functions of the modules associated with different etiologies varied. Abnormal formation of extracellular matrix was overlapping among five etiologies. The change in cytoskeleton organization was specifically detected in dilated cardiomyopathy. The activation of the Wnt receptor signaling pathway was limited to hypertrophic cardiomyopathy. The change in nucleosome and chromatin assembly was associated with only familial cardiomyopathy. Germ cell migration and disrupted cellular calcium ion homeostasis were solely detected in ischemic cardiomyopathy. The change in the metabolic process of glucose and triglyceride was detected in only post-partum cardiomyopathy.

**CONCLUSION:**

These results indicate that the pathogenesis of heart failure arising from different etiologies varies, which may provide molecular evidence supporting etiology-based treatment for heart failure patients.

## INTRODUCTION

Heart failure is the end stage of various heart diseases, including dilated cardiomyopathy, hypertension, hypertrophic cardiomyopathy, and myocardial infarction. Patients with heart failure suffer from a high mortality and a poor prognosis. Worldwide, the mortality of patients within 5 years of chronic heart failure is greater than 50% 1,2. Recently, the incidence of heart failure has been increasing due to improvement in the treatment of underlying diseases, changes of life style, and the aging of the population 3-5. Thus, heart failure is a very serious global public health problem in the 21^st^ century, and there is an urgent need to improve the outcome of such patients 6,7.

Currently, patients with chronic heart failure arising from different etiologies are given the same treatment in clinical practice, and the differences in etiology are ignored 6,7. Although the clinical presentation of heart failure has a similar appearance among such cases, the prognoses are different, suggesting that the pathogeneses of heart failure arising from different etiologies are different. This implies that the etiologies should be taken into consideration when deciding on treatment options, and etiology-based treatments should be given to patients with heart failure.

The purpose of this study was thus to elucidate whether the pathogeneses of various types of heart failure arising from different etiologies is different. The pathogenesis of diseases is usually not determined by a single gene but by the interactions among multiple genes forming a pathogenetic network, which is characterized by determining changes in the gene expression profile 8-11. In a pathogenetic network, some gene products have similar or the same functions. They are, thus, located in the same functional unit of the network, called a module, and work together to carry out their biological functions 12. A microarray globally detects the expression profile of the genome and is helpful for uncovering the pathogenesis of diseases. In this study, microarray data from the myocardial tissues of patients with heart failure arising from different etiologies were thus compared with those from normal controls to identify the differentially expressed genes (DEGs). Then, we constructed a protein-protein interaction (PPI) network of the DEGs and analyzed the modules of each PPI network. Finally, we explored the functions of these modules.

## MATERIALS AND METHODS

### Microarray data

GSE1145 was downloaded from the Gene Expression Omnibus (GEO) database. The samples contained in GSE1145 were myocardial tissues from patients with heart failure arising from five different etiologies, including 12 samples arising from dilated cardiomyopathy (DCM), 5 from familial cardiomyopathy (FCM), 5 from hypertrophic cardiomyopathy (HCM), 20 from ischemic cardiomyopathy (ISCM), and 4 from post-partum cardiomyopathy (PPCM), as well as 11 normal controls. The myocardial samples of heart failure were collected from patients undergoing cardiac transplantation whose failure arose from the different etiologies mentioned above. The normal control myocardial samples were collected from normal organ donors whose hearts could not be used for transplants. The platform used was GPL570 [HG-U133_Plus_2] Affymetrix Human Genome U133 Plus 2.0 Array, which contains a total of 54,675 probes. These probes correspond to 20,283 genes. When multiple probes corresponded to one gene, the variance of the expression levels of the probes was averaged as the expression value of the gene.

### Data preprocessing

The raw data from the original CEL files were preprocessed and transformed into expression values by Affy, an R package. Then, the expression values were normalized using the robust multiarray averaging (RMA) algorithm, and the missing data were filled in using the k-Nearest Neighbor (KNN) algorithm 13,14. Box plots were drawn before and after the normalization to estimate the degree of normalization 15,16.

### Analysis of DEGs

Limma, a package of R, was applied to identify the DEGs between each etiology and the normal controls 10. The statistical method that we used was the empirical Bayes method, which was implemented in limma 17. The threshold for the DEGs was set as an adjusted *p*-value <0.05, and the fold change values ≥2 16.

### Construction of the PPI network

Search Tool for the Retrieval of Interacting Genes (STRING, http://string-db.org/), a database containing both direct (physical) and indirect (functional) associations of protein interactions, was used to predict the interactions between the identified DEGs and to construct the protein-protein interaction (PPI) network 18.

### Analysis of the modules in the PPI network

Modules are functional units of a network. Genes with a similar or the same function make up a certain module. Here, Cytoscape was used to visualize the PPI network and the molecular complex detection (MCODE), a plugin of Cytoscape, was used to identify the modules in each network. The parameters of MCODE were as follows: the degrees of each node in a module were no less than 2, and subgraphs of each node were greater than 2 12.

### Enrichment analysis of the function of modules

The Database for Annotation, Visualization and Integrated Discovery (DAVID, https://david.ncifcrf.gov/) was applied to perform the functional enrichment analysis for the modules associated with the different etiologies 19. *P*<0.05 was set as the cut-off.

## RESULTS

### Data preprocessing and screening of the DEGs

**Figure 1:**
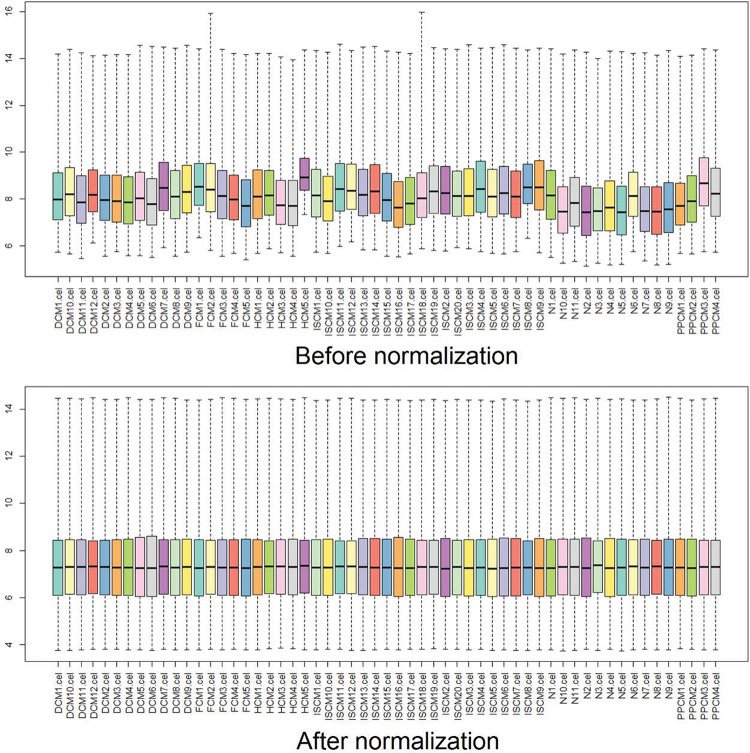
**Box plots for the expression value data before and after normalization.** The horizontal axis indicates the different samples, and the vertical axis represents the expression values of the genes. The black line in each box is the median of the expression value. Before normalization, the median gene expression value in each box was not at the same level. After normalization, the median of the expression value was almost on the same line, suggesting an excellent performance of the normalization.

**Figure 2:**
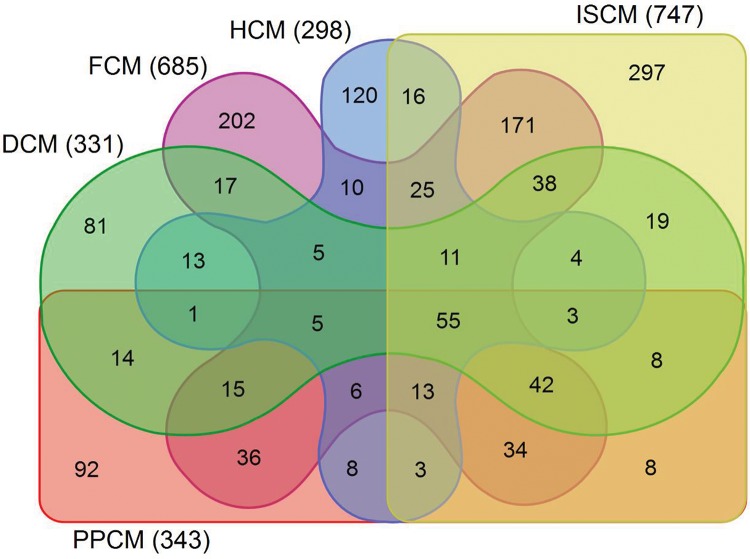
**Overlapping DEGs among the groups of different etiologies.** The Venn graph displays the number of differentially expressed genes overlapping among the different etiologies. The differentially expressed genes (DEGs) shown in the figure differ significantly in their expression value with an adjusted *p*-value <0.05 and a fold change >2. DCM, dilated cardiomyopathy; HCM, hypertrophic cardiomyopathy; FCM, familial cardiomyopathy; ISCM, ischemic cardiomyopathy; PPCM, post-partum cardiomyopathy.

After normalization, the median gene expression value in each box was approximately at the same level, which indicated an excellent degree of normalization (Figure 1). Compared with the normal controls, the numbers of DEGs in DCM, HCM, FCM, ISCM, and PPCM were different, namely, 331 (320 upregulated, 11 downregulated), 298 (198 upregulated, 100 downregulated), 685 (667 upregulated, 18 downregulated), 747 (731 upregulated, 16 downregulated), and 343 (306 upregulated, 37 downregulated), respectively (Table 1). The numbers of genes overlapping among the different etiologies are shown in Figure 2.

### PPI network construction

**Figure 3:**
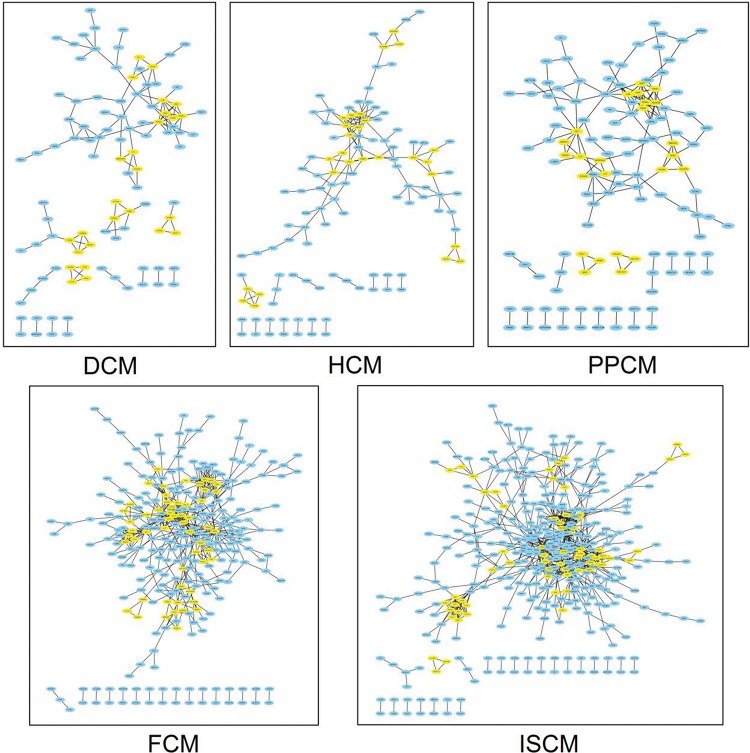
**The PPI network of each etiology.** The node represents the differentially expressed genes (DEGs), and the edge represents the interaction relationship among the products of the DEGs. The modules of each network are colored yellow. DCM, dilated cardiomyopathy; HCM, hypertrophic cardiomyopathy; FCM, familial cardiomyopathy; ISCM, ischemic cardiomyopathy; PPCM, post-partum cardiomyopathy.

Based on the DEGs screened previously, a PPI network of each etiology was constructed. The number of pairs of genes included in the PPI networks of DCM, HCM, FCM, ISCM, and PPCM were 128, 153, 507, 719, and 160, respectively. Figure 3 shows the PPI network visualized by Cytoscape.

### Analysis of the modules in the PPI network

**Figure 4:**
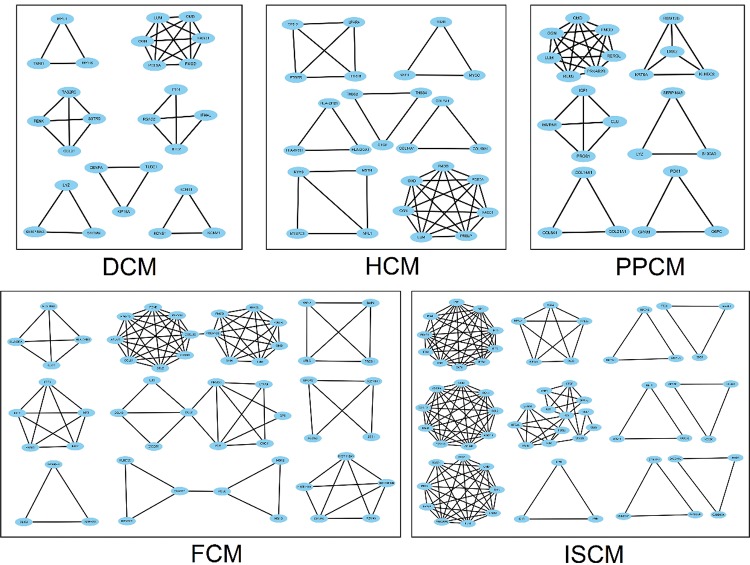
**Modules in the PPI network of the five different etiologies.** The node represents the module-related differentially expressed genes (DEGs), and the edge represents the interaction relationship among the products of the DEGs. DCM, dilated cardiomyopathy; HCM, hypertrophic cardiomyopathy; FCM, familial cardiomyopathy; ISCM, ischemic cardiomyopathy; PPCM, post-partum cardiomyopathy.

The number of modules in the PPI networks of DCM, HCM, FCM, ISCM, and PPCM was also different, namely, 7, 7, 9, 12, and 6, respectively (Figure 4). We analyzed the overlapping and unique module-related genes of the different etiologies and found that FMOD, LUM, OMD, and OGN were overlapping genes among the five etiologies, and they formed a module with overlap for all of the five etiologies. The numbers of unique module-related DEGs of DCM, HCM, FCM, ISCM, and PPCM were 8, 13, 29, 41, and 11, respectively, which was almost half of the total module-related DEGs (Table 2).

### Functional annotation of the modules

The functions of the modules in each PPI network were annotated using DAVID (Table 3). We found that the functions of the modules in each etiology were not exactly the same. The modules not only shared similarities but also exhibited differences. The modules related to immune/inflammatory response and the formation of the extracellular matrix were overlapping in the five etiologies. The genes in the modules related to immune response and inflammatory response were not equal among the different etiologies. However, FMOD, LUM, OMD, and OGN, which participate in the formation of the extracellular matrix, overlapped among the five etiologies. The modules related to cytoskeleton organization were detected specifically in heart failure arising from DCM, with increased KIF18A and TUBE1. The modules related to the Wnt receptor signaling pathway were limited to heart failure arising from HCM, with increased WIF1 and FRZB. The modules related to nucleosome and chromatin assembly were associated only with heart failure arising from FCM, with increased HIST1H2BN, CENPA, HIST1H1A and HIST1H2AK. The modules related to germ cell migration and disrupted cellular calcium ion homeostasis were solely detected in heart failure arising from ISCM, with increased CXCR4, CCL5, and CXCL12. The modules related to the metabolic process of glucose and triglycerides were detected only in heart failure arising from PPCM, with upregulated G6PC, GPAM, and PCK1. The genes in the modules related to the functions mentioned above are shown in Table 3.

## DISCUSSION

The immune response and inflammatory response play important roles in heart failure, leading to the development of this disease. Circulating inflammatory cytokines are elevated in heart failure and are used as predictors of clinical outcome 20-22. In our research, the immune/inflammatory response participated in heart failure arising from all five etiologies. Although the immune/inflammatory response was associated with heart failure induced by all five etiologies, the genes related to the immune/inflammatory response in the different etiologies were not exactly the same, which further indicates the differences in the pathogenesis in heart failure arising from these different etiologies. In addition to the immune/inflammatory response, our data showed that the formation of extracellular matrix overlapped among the types of heart failure arising from the five etiologies, and the genes related to this were the same. Cardiac remodeling is a key feature of heart failure, characterized by reduced myocytes and increased extracellular matrix, which finally result in cardiac fibrosis 23,24. The FMOD gene is a member of the family of small interstitial proteoglycans. The encoded protein may participate in the assembly of extracellular matrix due to interaction with type I and type II collagen fibrils 25. LUM, OMD, and OGN all belong to the family of small leucine-rich proteoglycans. LUM may regulate collagen fibril organization in the murine heart by coordinating multiple factors of collagen assembly, and OMD may reduce the diameter and change the shape of collagen fibrils by directly interacting with collagen 26,27. The upregulation of OGN may protect against cardiac fibrosis by inhibiting the proliferation and migration of cardiac fibroblasts 28. According to our results, we should take the immune/inflammatory response and cardiac fibrosis into consideration, and administering related treatments for patients with heart failure may be useful. FMOD, LUM, OMD, and OGN may be potential therapeutic targets. The activation of the neurohormonal and sympathetic systems has been demonstrated in heart failure, and blocking these pathways using angiotensin-converting enzyme inhibitors, angiotensin receptor blockers, and β-adrenergic blockers is useful to reduce the progression of heart failure and improve clinical outcomes 29-31. However, no evidence of abnormal neurohormonal and sympathetic systems was observed in our data. Considering that the samples used for GSE1145 were cardiac tissue rather than a single type of cardiac cell, we hypothesized that changes in the neurohormonal and sympathetic systems could occur in a certain cell type and that the expression level of related genes may be diluted when detected in cardiac tissue that is a mixture of multiple cardiac cell types.

About half of patients with heart failure present an enlarged heart and reduced cardiac pump function, which are accompanied by cytoskeletal changes. Such changes are not only the cause but also the consequence of reduced systolic function in patients with heart failure. The cytoskeleton forms a complex network that extends through the cytoplasm and connects the nucleus, the plasma membrane, and even the extracellular matrix. The cytoskeleton participates in the dilatation and contraction of the heart 32,33. DCM is a primary etiology of heart failure with cardiac dilatation and decreased cardiac function 34. We found that the changes in cytoskeleton organization were detected specifically in heart failure arising from DCM. KIF18A belongs to the kinesin superfamily of microtubule-associated molecular motors and regulates microtubule dynamics 35. TUBE1 is a member of the tubulin superfamily and plays a central role in the organization of microtubules 36. These results indicate that the cytoskeleton may play an important role in the pathogenesis of heart failure resulting from DCM. We should, thus, focus on the changes in the cytoskeleton in patients with heart failure arising from DCM. Administering related treatment to these patients may reverse cytoskeletal abnormalities and KIF18A and TUBE1 may be potential therapeutic targets.

Wnt signaling is involved in various biological processes. Previous studies have shown that such signaling is reactivated under pathological conditions but mostly remains silent in a normal state. Increasing evidence suggests that Wnt signaling participates in the progression of heart failure and is related to adverse cardiac remodeling 37,38. HCM is a primary myocardial disease that commonly causes thickening of the myocardium 39. In our study, the activation of the Wnt receptor signaling pathway was limited to heart failure arising from HCM. The protein encoded by WIF1 functions to inhibit Wnt signaling and may impair the function and structure of the heart 40. FRZB is a type of secreted Wnt antagonist that may inhibit fibrosis in vitro 41. This result indicates that Wnt signaling may be extremely important for the pathogenesis of heart failure resulting from HCM. Wnt signaling-related treatment may have potential benefits to patients with heart failure arising from HCM, and WIF1 and FRZB may be potential therapeutic targets.

Epigenetic regulation plays an important role in various pathological and physiological conditions. The main mechanisms of epigenetic regulation include DNA methylation and histone modifications, which influence gene expression by affecting the assembly of the nucleosome and chromatin. Studies have shown that epigenetic regulatory mechanisms participate in heart failure and modulate the expression of multiple genes that are essential for the development of heart failure 42. FCM is a genetic disorder that is difficult to recognize until advanced phenotypic manifestations occur. The late phenotypes of FCM, such as an enlarged atrium, are subtle in comparison with those of HCM and DCM 43. Our data showed that changes in nucleosome and chromatin assembly were associated only with heart failure arising from FCM. HIST1H2BN and HIST1H2AK encode a replication-dependent histone, which belongs to the histone H2B family. They are both linked to histone H1 and participate in the compaction of chromatin into higher order structures in transcriptional regulation 44,45. CENPA encodes a centromere protein that contains a histone H3-related histone fold domain. The protein encoded by CENPA is proposed to be a component of a modified nucleosome in which it replaces 1 or both copies of the conventional histone H3 46. HIST1H1A encodes a replication-dependent histone that is a member of the histone H1 family, which interacts with linker DNA between nucleosomes and functions in the compaction of chromatin into higher order structures 47. The results suggest that epigenetic regulation is important for heart failure arising from FCM. Epigenetic changes in FCM, such as increased HIST1H2BN, CENPA, HIST1H1A and HIST1H2AK, may thus be useful for distinguishing FCM from HCM and DCM and may also be a useful therapeutic target for patients with heart failure arising from FCM.

ISCM is caused by myocardial infarction, which eventually develops into heart failure. It is characterized by a loss of cardiomyocytes and the disruption of cellular calcium ion homeostasis in the infarcted region, which leads to ventricular reconstruction and cardiac dysfunction. Stem cell-based treatments in heart failure have been tested in many trials, and the results are promising 48. Our data showed that germ cell migration was solely detected in heart failure arising from ISCM, suggesting that a stem cell-based treatment may be especially beneficial for ISCM-induced heart failure. CXCR4 encodes a CXC chemokine receptor, which has 7 transmembrane regions and is located on the cell surface. CXCR4 can induce stem cell migration through the FAK/PI3K/Akt and GSK3β/β-catenin pathways 49. The protein encoded by CXCL12 functions as the ligand for the G-protein coupled receptor and can bind to CXCR4 and regulate the migration of stem cells 50-52. Calcium ion homeostasis is broken in ISCM. Previous studies have indicated that Ca^2+^ signaling is related to the regulation of cardiac remodeling and turnover 53. CCL5 is one of several chemokine genes clustered on the q-arm of chromosome 17 and can activate calcium signals through a multistep cascade 54. CXCR4 protein expression is influenced by extracellular calcium and, thus, may enhance stem cell migration 55. CXCL12 stimulates the release of intracellular calcium in a dose-dependent manner. CXCL12-stimulated epithelial cell migration can be abrogated by intracellular calcium chelation 56. Based on our study, a disrupted cellular calcium ion homeostasis was solely detected in ISCM. These results indicate that the regulation of disrupted cellular calcium ion homeostasis may have potential advantages for treating heart failure arising from ISCM, and CXCR4, CCL5, and CXCL12 may be potential therapeutic targets. However, CXCR4 and CXCL12 are also involved in germ cell migration, and further studies are required to clarify the potential efficacy of the CXCR4 and CXCL12 in clinical settings.

Heart failure is accompanied by energy metabolic remodeling, which in turn exacerbates heart failure. Free fatty acids (FFAs) are the main substrate for the normal heart to produce ATP. Approximately 60%-90% of ATP is derived from the aerobic oxidation of FFAs. Upon the development of heart failure, the substrate utilization changes. The aerobic oxidation of FFAs diminishes, and the uptake of glucose increases. The metabolic pattern also changes from the aerobic oxidation of FFAs to anaerobic glycolysis 57-59. PPCM is a secondary myocardial disease in women with left ventricular failure and occasionally right ventricular failure. It occurs more frequently during the last month of pregnancy or within the first 6 months after delivery 60. Our study found that changes in the metabolic process of glucose and triglycerides were only detected in PPCM-induced heart failure, indicating that energy metabolic remodeling may be more important in PPCM-induced heart failure. G6PC is a multisubunit integral membrane protein that is composed of a catalytic subunit and transporters for glucose. The protein encoded by G6PC is a key enzyme in glucose homeostasis and catalyzes the hydrolysis of D-glucose 6-phosphate to D-glucose and orthophosphate 61. GPAM is an isoform of glycerol-3-phosphate acyltransferase and is located on the outer mitochondrial membrane. GAPM is required to catalyze de novo synthesized fatty acids into triacylglycerol and, thus, to divert them away from oxidation 62. PCK1 is a key player in the initial step of gluconeogenesis and can decrease circulating free fatty acids 63. Energy metabolic remodeling related molecules, such as G6PC, GPAM, and PCK1, may emerge as potential therapeutic targets for these patients.

The major strength of this study was that based on the microarray dataset, we showed different pathogeneses of heart failure arising from different etiologies and found the similarities and differences in the DEGs among the different types of heart failure. However, because there are only 4 or 5 samples for FCM, HCM and PPCM, our results of these three groups may have been due to chance and must be confirmed by increasing the sample size.

Our results indicate that the pathogenesis of heart failure arising from different etiologies not only shares similarities but also exhibits differences, which may provide molecular evidence supporting the concept that etiology-based treatment is required for patients with heart failure.

## AUTHOR CONTRIBUTIONS

Ma A, Yang G and Wang T participated in the design of the study. Yang G and Lu J carried out the study. Yang G and Chen S analyzed the data. Yang G and Wang T drafted the manuscript.

## Figures and Tables

**Table 1:** The number of DEGs in the different etiologies.

Etiologies	DEGs	Upregulated	Downregulated
DCM	331	320	11
HCM	298	198	100
FCM	685	667	18
ISCM	747	731	16
PPCM	343	306	37

**Table 2:** Overlapping and unique DEGs in the modules of the different etiologies.

Etiologies	Total	Genes
Overlapping	4	FMOD LUM OMD OGN
HCM 27	13	WIF1 MSTN PRELP HLA-DPB1 MYBPC1 MYOC HTR2B THBS2 FRZB HLA-DQA1 PTGFR THBS4 COL10A1
DCM 8	8	TNNI1 TUBE1 KCNV1 TAS2R5 KCNB1 SSTR5 KCNS1 KIF18A
FCM 55	29	HIST1H2AK POLK CLIC2 ABCG2 CX3CR1 CD74 HIST1H2BN HLA-DRA ZEB1 HLA-DMA MSH3 GABRR3 CCL18 ALDH1A1 GPR18 RBM43 SNAI2 IL10 CCL2 HIST1H1A PROM1 PIK3CG MSH2 EPCAM SCARF1 GNG11 GP5 GABRG3 KITLG
ISCM (67)	41	SPARC FOS RGS2 RPL26 CXCR4 TTC8 HTR2A VCAM1 EIF1AY CD69 FAM134B HMGN3 NDC80 IFITM1 GADD45G FST RPS4Y1 HMGN1 PLEC HMGN2 BBS10 ARL14 SPC25 XAF1 GADD45A C11orf70 RAP1B RAB33B PRKX KIF20A GHR LIFR SESN1 RGS1 BET1 NSA2 IFNK CCL4 CCDC111 IFI27 BBS5
PPCM 24	11	MMRN1 IGF1 CLU PROS1 COL21A1 DKK2 KRT6A PCK1 REM2 GPAM G6PC

The numbers in parentheses indicate the total number of module-related DEGs.

**Table 3:** The annotated functions of the modules in the PPI network of each etiology.

Etiology	Description	*p*-value	Gene
**DCM**			
	immune response	0.0099	RSAD2, IFI44L
	inflammatory response	5.75E-4	S100A9↓, SERPINA3↓, LYZ
	cytoskeleton-dependent intracellular transport	0.0077	MYL1, MYH6↓
	muscle contraction	0.0225	MYL1, MYH6↓
	muscle organ development	0.0310	MYL1, MYH6↓
	cytoskeleton organization	0.0010	CENPA, KIF18A, TUBE1
	cell cycle process	0.0017	CENPA, KIF18A, TUBE1
	potassium ion transport	1.39E-4	KCNS1, KCNB1, KCNV1
	extracellular matrix	1.95E-5	FMOD, LUM, OMD, OGN
**HCM**			
	muscle organ development	7.19E-4	MYL1, MSTN, MYH6↓
	muscle contraction	3.78E-4	MYBPC1↓, MYL1, MYH6↓
	cytoskeleton-dependent intracellular transport	0.0115	MYL1, MYH6↓
	Wnt receptor signaling pathway	0.0098	WIF1, FRZB
	immune response	0.0026	HLA-DRB1, HLA-DPB1, HLA-DQA1
	extracellular matrix	1.95E-5	FMOD, LUM, OMD, OGN
**FCM**			
	immune response	0.0258	CCL21, P2RY14, CCL5, CXCL12, HLA-DRB1, HLA-DMA, CD74, HLA-DRA
	nucleosome assembly	2.31E-7	HIST1H2BN, CENPA, HIST1H1A, HIST1H2AK
	chromatin assembly	2.57E-7	HIST1H2BN, CENPA, HIST1H1A, HIST1H2AK
	positive regulation of protein amino acid phosphorylation	0.0196	BMP4, KITLG
	inflammatory response	7.03E-4	CCL2, IL10, CCL18, F2R
	negative regulation of programmed cell death	9.40E-4	PIK3CG, CCL2, IL10, F2R
	JAK-STAT cascade	0.0228	CCL2, F2R
	anion transport	0.0210	GABRG3, CLIC2
	extracellular matrix	1.95E-5	FMOD, LUM, OMD, OGN
**ISCM**			
	immune response	1.68E-5	RGS1, CCL21, CXCR4, P2RY14, CCL5, CXCL12
	cellular calcium ion homeostasis	0.0048	CXCR4, CCL5, CXCL12
	germ cell migration	0.0071	CXCR4, CXCL12
	patterning of blood vessels	0.0124	CXCR4, CXCL12
	inflammatory response	0.0146	CCL21, CXCR4, CCL5/FOS, CCL4, F2R
	ncRNA processing	0.0409	RPL26, NSA2
	regulation of cell proliferation	0.0027	VCAM1, BMP4, SPARC, F2R, HTR2A
	microtubule cytoskeleton organization	0.0216	SPC25, NDC80
	cellular response to stress	0.0017	GADD45G, SESN1, GADD45A
	regulation of cell cycle	0.0483	GADD45G, GADD45A
	extracellular matrix	1.95E-5	FMOD, LUM, OMD, OGN
**PPCM**			
	response to wounding	5.98E-5	CLU, IGF1, MMRN1, PROS1
	blood coagulation	0.0225	MMRN1, PROS1
	anti-apoptosis	0.0450	CLU, IGF1
	inflammatory response	5.75E-4	S100A9↓, SERPINA3↓, LYZ
	triglyceride metabolic process	9.87E-6	G6PC, GPAM, PCK1
	glucose metabolic process	0.0225	G6PC, PCK1
	extracellular matrix	1.95E-5	FMOD, LUM, OMD, OGN

The downregulated genes are marked “↓”, and the other genes are upregulated.
